# Primary Amenorrhea, Aplastic Uterus, and a Functional Vagina: A Rare Case of Mayer-Rokitansky-Küster-Hauser Syndrome

**DOI:** 10.1155/2019/3409548

**Published:** 2019-05-28

**Authors:** Gregory W. Kirschen, Natalie Semenyuk

**Affiliations:** ^1^Medical Scientist Training Program, Stony Brook Medicine, USA; ^2^Albert Einstein College of Medicine, Montefiore Medical Center, USA

## Abstract

**Background:**

Müllerian agenesis, also known as Mayer-Rokitansky-Küster-Hauser syndrome (MRKHS), a failure of female urogenital development, typically results in a completely stenotic or rudimentary dimple vagina, both of which are generally nonfunctional in adulthood without mechanical dilation or surgical reconstruction.

**Case:**

A 20-year-old Tanner stage V heterosexual woman with normal sexual function since coitarche presented with a chief complaint of primary amenorrhea. She was found to have aplastic uterine buds, absent endometrium/cervix, normal ovaries, and an unusually well-developed lower vagina, a rare presentation of MRKHS. We discuss mechanisms by which the anomaly may have arisen.

**Summary & Conclusion:**

This case thus expands the clinical presentation of MRKHS to include a normal appearing vagina with intact sexual function from first sexual encounter, raising interesting questions about the basic underlying embryology.

## 1. Introduction

The Müllerian ducts are paired tubes abutting the urogenital ridge that give rise to the upper portion of the female reproductive tract. Failure of proper development of the upper female urogenital tract results in a wide spectrum of anatomical abnormalities of the gynecological and urological systems. Müllerian agenesis, also known as Mayer-Rokitansky-Küster-Hauser syndrome (MRKHS), represents a failure of urogenital development with an estimated incidence of 1/4500 live female births, characterized by absence or aplasia of the uterus, cervix, and/or upper vagina without or with associated urological and other organ system involvement (MRKHS type I versus type II, respectively) [1].

Depending on the particular structures affected and the severity of involvement, such abnormalities may be detected at birth or may go clinically unnoticed until there is absence of menarche or complaints of dyspareunia/sexual dysfunction with attempted sexual activity [2]. Vaginal agenesis during embryological development often leads to complete absence of the vagina if not the remnant of a small vaginal dimple in adults [2]. Here, we report an unusual case of MRKHS in an otherwise healthy, previously sexually active woman who presented at age 20 with primary amenorrhea and was found to have aplastic uterine buds, absent endometrium, and absent cervix, but with a well-developed lower vagina with intact sexual function. This case highlights a unique variant of MRKHS with a normal appearing vagina and intact sexual function without any medical or surgical intervention, expanding the clinical presentation of this condition.

Informed consent was obtained from the patient to use her medical information for this case report.

## 2. Case

A 20-year-old woman from Honduras presented on 3/20/2018 with her adoptive mother for a normal gynecological annual exam with a chief complaint of never having a menstrual period. She had lacked access to gynecological care in Honduras and had thus never been previously evaluated by a gynecologist. Her first sexual encounter was at age 17 and she reported having penetrative vaginal intercourse on a few occasions, although she was not currently sexually active at the time of evaluation. Previous sexual history included one male partner, condom use for protection against sexually transmitted infections, and good sexual function. Importantly, she had never experienced poor sexual satisfaction or dyspareunia. Her known family history was limited but was significant for a cousin who had also never had a menstrual period and a maternal grandmother who had had a brain tumor of unspecified type. She took no medications, and her only allergy was penicillin, with no reaction documented. She denied tobacco or illicit drug use and used alcohol occasionally. She denied any breast lumps, masses, nipple discharge, breast pain, excessive facial/body hair, abdominal/pelvic pain, genital lesions, rashes, or pruritus.

On physical exam, her vital signs were within normal limits and her body mass index (BMI) was 25.51. She was alert and oriented and in no acute distress. Neck was without lymphadenopathy or thyromegaly. Breasts were well-developed with no masses, tenderness, or discharge, Tanner stage V. Abdomen was soft and nontender. Gynecological exam showed no lesions and normal adult female pubic hair pattern, Tanner stage V. The cervix was unable to be palpated or visualized. The uterus was notpalpable and no adnexal masses were appreciated. The vagina was normal in appearance, measured 8 cm, was two fingerbreadths in diameter, and was without bleeding or discharge. The urethra was normal in appearance. The patient was cooperative, with appropriate mood and affect.

**Figure 1:**
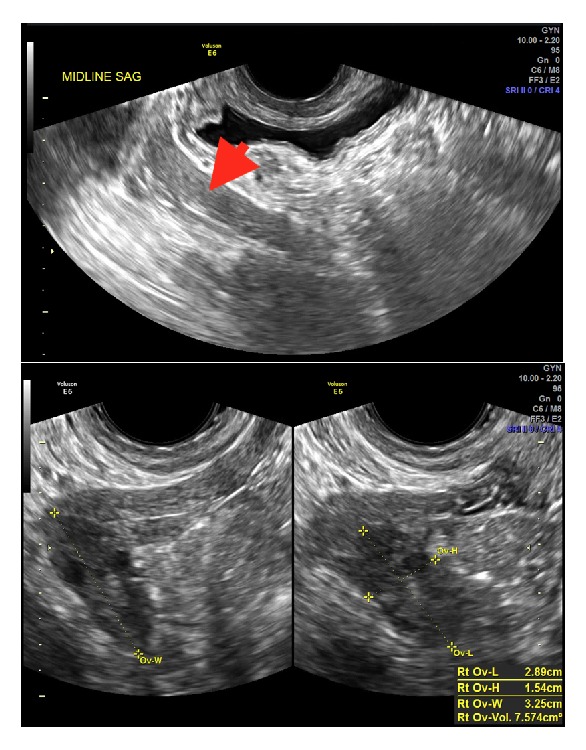
Ultrasound demonstrating bilateral aplastic left (top) and right (bottom) hemiuteri with normal-sized ovaries.

In light of the patient's presentation, transabdominal/transvaginal ultrasound was performed on 3/22/2018, which revealed bilateral remnant uteri with no endometrium appreciated (Figure 1). The right remnant of the uterus was more prominent compared to the left. The cervix was not imaged. Follicle-containing ovaries were imaged transvaginally and transabdominally and were normal in size and appearance bilaterally, definitively excluding the presence of testes and effectively ruling out congenital androgen insensitivity syndrome (CAIS). There were no ovarian cysts or masses imaged. No fluid was appreciated in the cul de sac.

A follow-up MRI of the pelvis without and with contrast performed on 4/4/2018 demonstrated absence of the cervix, bilateral homogeneous enhancing uterine buds within the pelvis measuring 3.0 × 1.7 × 2.3 cm on right, 2.9 × 1.7 × 2.6 cm left. The upper one-third of the vagina was absent, while the lower two-thirds were present. The right ovary was of normal size, measuring 2.8 × 1.4 × 1.6 cm, and with multiple normal ovarian follicles. The left ovary was also of normal size measuring 2.1 × 1.5 × 1.9 cm, with multiple normal ovarian follicles and 1 cm left corpus luteum. Trace physiological free fluid was identified. No enlarged lymph nodes were noted. The bladder was unremarkable. The urethra was notable for 2 adjacent tiny cystic structures abutting the right posterior aspect of the external urethral meatus measuring 0.6 cm and 0.3 cm, respectively, most compatible with tiny Skene gland cysts. Together, the findings of primary amenorrhea, normal ovaries and female secondary sexual characteristics, and aplasia of the uterus with absence of the cervix and upper one-third of the vagina, were consistent with a diagnosis of MRKHS.

A subsequent biochemical analysis was performed to further support the diagnosis of MRKHS. Levels of estriol, follicle-stimulating hormone (FSH), luteinizing hormone (LH), total testosterone, and anti-Müllerian hormone (AMH) were all within normal limits, again consistent with the diagnosis of MRKHS (Table 1). The patient declined karyotype analysis due to financial limitations.

Because of the well-known association between MRKHS and anatomical abnormalities of the urological system [2], a renal ultrasound was performed on 4/20/2018, which demonstrated normal bilateral kidneys, no evidence of hydronephrosis, and no evidence of contour deforming mass or shadowing renal calculus. Bilateral ureteral jets were demonstrated on color Doppler, and no distal hydroureter was seen. Thus, our patient displayed characteristics of MRKHS type I.

Upon hearing the diagnosis, our patient was anxious, especially with regard to future reproductive prospects; however she was counseled for 45 minutes with her stepmother/guardian present regarding the implications of the diagnosis and reproductive options such as use of a surrogate to carry a pregnancy for her. She expressed gratitude at the end of the encounter for the information and services provided and was offered follow-up as needed.

Upon last follow-up (August, 2018), the patient was doing well at age 21, living with her supportive stepmother, and not sexually active.

## 3. Summary & Conclusion

MRKHS represents a spectrum of urogenital anomalies arising from failure of the upper female reproductive tract (Müllerian duct derivatives) to properly form during embryogenesis. In cases of MRKHS type I, patients exhibit varying degrees of congenital aplasia of the uterus and upper vagina, without extragynecological involvement and with normal secondary sexual characteristics [2]. Cases of MRKHS type II involve renal, vertebral, auditory, and/or cardiac defects in addition to the aforementioned gynecological anomalies.

Regardless of subtype, the majority of patients afflicted by MRKHS exhibit vaginal aplasia, with a rudimentary vaginal dimple measuring between 1 and 4 cm that, untreated, may preclude penile-vaginal intercourse or lead to sexual dysfunction and/or dyspareunia [2–4], although not in all cases. It is important to point out that many women with MRKHS or CAIS report adequate sexual function, including penile-vaginal intercourse, at baseline, and nonsurgical or surgical approaches may be of benefit only to select candidates who experience sexual dysfunction attributable to an anatomical abnormality [5]. Likewise, a woman's actual vaginal length may be a poor surrogate for sexual experience, and practitioners should not assume that a short vaginal length implies inadequate sexual function. Rather, women may perceive that their vaginas are “too small,” which may lead to psychological stress that must be addressed by the provider but which may not accurately reflect potential for satisfying sexual experience [5]. Management for those who do desire intervention is aimed at improving sexual function and ranges from nonsurgical to surgical, including serial vaginal dilation (first-line) and surgical neovagina construction (second-line) [6]. Treatment of these women must focus on psychological preparedness for sexual activity and any fears or anxiety that the women may suffer. As one of the reviewers pointed out, there are two valuable resources that practitioners taking care of patient with MRKHS should be aware of, the support group BEAUTIFUL YOU (https://www.beautifulyoumrkh.org) and Accord Alliance (https://www.accordalliance.org), an organization that lists a number of support groups for people affected by disorders of sexual development. We will counsel our patient about these resources, and we encourage others to do the same.

Here, we report a case of a woman with MRKHS with a well-formed lower vagina and satisfactory sexual function since onset of first intercourse and with a sexual history of only a handful of previous penetrative sexual encounters, whose only complaint was primary amenorrhea. One limitation of our report is that we did not have access to a vaginal length before onset of intercourse and are thus unable to state unequivocally whether sexual intercourse played a role in vaginal expansion, although we feel this possibility is less likely given her sexual history. In addition, we were unable to confirm the patient's karyotype, which would have provided further support of the diagnosis of MRKHS over CAIS, although presence of pubic hair, sonographic and MRI evidence of ovaries, and normal female hormonal profiles in the absence of a formed uterus and upper vagina all favor MRKHS (Figure 1 and Table 1). Nevertheless, clinical features and imaging, particularly MRI, which was also obtained in this case, are sufficient to make the diagnosis of MRKHS [7]. However, in equivocal cases unlike ours, a karyotype should be obtained to confirm the diagnosis.

In any case, we hypothesize that in our patient's case, the urogenital sinus, responsible for giving rise to the lower two-thirds of the vagina, may have undergone unusually extensive proliferation during the 4th and 5th months of embryonic life, giving rise to a more elongated and well-canalized vagina than what would typically develop. This could compensate for the contraction and stenosis that would otherwise occur in the face of an aplastic upper vagina (uterovaginal primordium). While most cases of MRKHS are sporadic, a subgroup of patients has been shown to harbor mutations in* WNT *family genes [8]. Given the well-established role of WNT signaling in cellular proliferation, dysregulation of this pathway in our patient may have contributed to her phenotype, although this remains unknown at present. One historical feature consistent with a genetic cause of MRKHS is the patient's relative who also exhibited primary amenorrhea. However, further evaluation and work-up would have been necessary to confirm the diagnosis in her relative, and genetic testing of either of these women could potentially reveal an underlying polymorphism or mutation (though this can be costly).

As an alternative explanation, our patient may have undergone a more prolonged period of lower vaginal growth during embryogenesis than typically occurs, compensating for upper reproductive tract aplasia. The normal resting adult human vagina is known to vary in linear length of ~40-100 mm, although the precise genetic or environmental factors determining this final length remain to be completely understood [9]. Perhaps cases like the one we present will serve to spark new interest in revisiting the complex embryological processes underlying female reproductive tract development.

In summary, here we present an atypical case of MRKHS with a well-developed lower vagina in the absence of a cervix and aplastic uterine buds, in a sexually active woman with no sexual dysfunction. This case demonstrates that vaginal agenesis/dimpling and poor sexual function are not requisite features of MRKHS, providing evidence that contradicts traditional views of Müllerian development.

## Figures and Tables

**Table 1:** Biochemical evidence of female phenotype.

Serum marker	Patient value (ng/mL)	Reference range (ng/mL)
Estriol	208.1	43.8—211.0 (Luteal phase)
Follicle-stimulating hormone	1.6	1.7—7.7 (Luteal phase)
Luteinizing hormone	4.8	1.0-11.4 (Luteal phase)
Testosterone (total)	27	8—48
Anti-Müllerian hormone	2.57	1.23-11.51 (females 20-25 years)

## Abbreviations

MRKHS: Mayer-Rokitansky-Küster-Hauser syndrome
MRI: Magnetic resonance imaging.
